# Characterization of the chloroplast genome of *Gleditsia* species and comparative analysis

**DOI:** 10.1038/s41598-024-54608-6

**Published:** 2024-02-21

**Authors:** Feng Xiao, Yang Zhao, Xiurong Wang, Xueyan Jian

**Affiliations:** 1 Institute for Forest Resources and Environment of Guizhou, Key Laboratory of Forest Cultivation in Plateau Mountain of Guizhou Province, College of Forestry, Guizhou University, Guiyang, 550025 Guizhou China; 2 College of Continuing Education, Yanbian University, Yanji, 133002 Jilin China

**Keywords:** Evolution, Genetics, Plant sciences

## Abstract

The genus *Gleditsia* has significant medicinal and economic value, but information about the chloroplast genomic characteristics of *Gleditsia* species has been limited. Using the Illumina sequencing, we assembled and annotated the whole chloroplast genomes of seven *Gleditsia* species (*Gleditsia sinensis*, *Gleditsia japonica* var. *delavayi* (*G. delavayi*), *G. fera*, *G. japonica*, *G. microphylla*, *Fructus Gleditsiae Abnormalis* (Zhū Yá Zào), *G. microphylla* mutant). The assembled genomes revealed that *Gleditsia* species have a typical circular tetrad structure, with genome sizes ranging from 162,746 to 170,907 bp. Comparative genomic analysis showed that most (65.8–75.8%) of the abundant simple sequence repeats in *Gleditsia* and *Gymnocladus* species were located in the large single copy region. The *Gleditsia* chloroplast genome prefer T/A-ending codons and avoid C/G-ending codons, positive selection was acting on the *rpoA*, *rpl20*, *atpB*, *ndhA* and *ycf4* genes, most of the chloroplast genes of *Gleditsia* species underwent purifying selection. Expansion and contraction of the inverted repeat (IR)/single copy (SC) region showed similar patterns within the *Gleditsia* genus. Polymorphism analysis revealed that coding regions were more conserved than non-coding regions, and the IR region was more conserved than the SC region. Mutational hotspots were mostly found in intergenic regions such as “*rps16*-*trnQ*”, “*trnT*-*trnL*”, “*ndhG*-*ndhI*”, and "*rpl32*-*trnL*” in *Gleditsia*. Phylogenetic analysis showed that *G. fera* is most closely related to *G. sinensis,G. japonica* and *G. delavayi* are relatively closely related. Zhū Yá Zào can be considered a bud mutation of the *G. sinensis.* The albino phenotype of *G. microphylla* mutant is not caused by variations in the chloroplast genome, and that the occurrence of the albino phenotype may be due to mutations in chloroplast-related genes involved in splicing or localization functions. This study will help us enhance our exploration of the genetic evolution and geographical origins of the *Gleditsia* genus.

## Introduction

The plants in the genus *Gleditsia*, mainly distributed in central and Southeast Asia and North and South America, they have been used as local and traditional medicines in many regions, particularly in China^1^. The genus recognized 13 species^2^. There are 6 species and 2 varieties of *Gleditsia* plants in China, including *Gleditsia sinensis*, *G. australis*, *G. fera*, *G. japonica*, *G. microphylla*, *Gleditsia japonica* var. *delavayi* (*G. delavayi*), *Gleditsia japonica* var. *velutina* (*G. velutina*), and 1 species (*G. triacanthos*) which is introduced^3,4^. *G. sinensis* (Fam.: *Leguminosae*; Gen.: *Gleditsia*), deciduous tree or shrub-like, contains both diploids and having 2n = 28 chromosomes^5^. *G. sinensis* is widely distributed in China and is resistant to drought, cold, pollution, and has strong stress resistance, it integrates medicinal, edible, chemical, material, and ornamental purposes^6^. *Fructus Gleditsiae Abnormalis* (Zhū Yá Zào)*,* it is the dried and sterile fruit of *G. sinensis*, there are significant differences in the morphology, structure and composition of *G. sinensis* and Zhū Yá Zào^7^, Li et al.^8^ suggested that it should be a variant of *G. sinensis*. However, the way in which Zhū Yá Zào came into being is still unknown.

Identifying the genetic evolutionary relationship between *Gleditsia* is the key to distinguish varieties. Chloroplast gene sequences (*ndhF* and *rpl16*) are selected to test biogeographic hypotheses, there is a fundamental division of the genus *Gleditsia* into three clades^9^. Based on the ITS sequence, Schnabel^10^ conducts a systematic evolutionary study on the 11 species of *Gleditsia* and the results shows that the *Gleditsia* and *Gymnocladus* appear to have originated in eastern Asia during the Eocene. Xing^11^ selected three fragments of chloroplast DNA, *MatK*, *PsbA-trnH*, and *TrnL-F* to establish a phylogenetic tree of *Gleditsia*, the results shows that the *Gymnocladus* has a longer evolutionary time than the *Gleditsia*. The complete chloroplast genome contains a large amount of genetic information and is highly conserved. The self-replication and evolution of its genome remain relatively independent of species. The use of DNA barcodes of the chloroplast genome will help identify varieties and resources^12^. In recent years, with the development of high-throughput sequencing technology, an increasing number of plant chloroplast genome DNA sequences have been obtained. However, in the *Gleditsia* genus*,* only the chloroplast genomes of *G. sinensis* and *G. japonica* have been reported so far^13,14^, this limits our understanding of the genetic evolution of *Gleditsia.* The development of genomic resources for *Gleditsia* can also assist in molecular breeding studies of this genus. The collected seven *Gleditsia* species (*Gleditsia sinensis*, *G. delavayi*, *G. fera*, *G. japonica*, *G. microphylla*, Zhū Yá Zào, *G. microphylla* mutant) and two *Gymnocladus* species (*Gymnocladus chinensis*, *Gymnocladus dioicus*) were sequencing, assembled and analyzed, complete chloroplast genomes were obtained. In this study, the seven species chloroplast genome of *Gleditsia* genus were sequenced, assembled and analyzed to study any common features or differences between species, which helps in the genetic breeding and molecular evolution of *Gleditsia*.

## Materials and methods

### Plant materials, genomic DNA isolation and genome sequencing

We collected various species seed of *Gleditsia* in China, including *G. sinensis* (Luodian City, Guizhou Province) (E 106.7379, N 25.2454), *G. fera* (Ceheng City, Guizhou Province) (E 105.9642, N 24.9327), *G. japonica* (Guiyang City, Guizhou Province) (E 106.9382, N 26.6969), *G. microphylla* (Zhijin City, Guizhou Province) (E 106.0246, N 26.5328), *G. japonica* var. *delavayi* (*G. delavayi*) (Xinyi City, Guizhou Province) (E 104.9381, N 25.1755). In addition, there were no seeds of Zhū Yá Zào, we directly collected the leaves of the plant Zhū Yá Zào (Tongren City, Guizhou Province) (E 108.0349; N 27.9852) (Fig. S1). *Gymnocladus chinensis* (Gen.: *Gymnocladus*) and *Gymnocladus dioicus* (Gen.: *Gymnocladus*) were collected as out group. After bringing various seeds back to the laboratory, then placed in the plant greenhouse incubator for cultivation. In the process of raising seedlings in the greenhouse, a albino mutant plant of *G. microphylla* was obtained (labeled *G. microphylla* mutant), which was characterized by albino whole plant, obvious dwarfing, and natural death after 1–1.5 months of growth. Taking single plant leaves of above all species for DNA extraction. Total DNA of each sample was isolated according to the instructions of the DNA extraction kit (EasyPure® Plant Genomic DNA Kit, Beijing Quanshijin Biological *Co.*, Ltd.). Nandrop 2000 (Thermo Fisher Scientific, Waltham, Massachusetts, USA) was used to determine the concentration and purity of the DNA. The DNA integrity was assessed by agarose gel electrophoresis. According to the Illumina standard protocol, total DNA was used to generate libraries after DNA extraction which were sequenced using the Illumina NovaSeq 6000 platform, and the sequencing read length was 150 bp.

All methods were performed on *Gleditsia* plants that were cultivated for the purposes of the experiments, including the collection of plant material for all analysis, and all relevant institutional, national, and international guidelines and legislation were complied with.

### Chloroplast genome assembly, annotation and sequence analysis

The raw data quality control was performed by fastp (v0.12.4)^15^. The clean paired-end reads were assembled with GetOrganelle (v1.7.6.1)^16^. The complete chloroplast genome was annotated in CPGAVAS2^17^ (http://www.herbalgenomics.org/cpgavas2/). The the transfer RNA (tRNA) genes were verified with tRNAscan-SE^18^. REPuter^19^ (https://bibiserv.cebitec.uni-bielefeld.de/reputer/) was used to find the sizes and locations of forward, reverse, palindromic, and complementary repeats. Simple sequence repeat (SSRs) were determined using a Perl script MISA (MIcroSAtellite identification tools), including mono-, di-, tri-, tetra-, penta-, and hexa-nucleotides, minimum numbers (thresholds) were 10, 5, 4, 3, 3, and 3, respectively. The chloroplast genome sequences were deposited in GenBank (Accession Numbers: OP722579–OP722582).

### Analysis of codon usage bias and selective pressures in the evolution

Extracted the full-length coding sequences, with an ATG start codon, a stop codon (TGA/TAG/TAA). The nucleotide compositions at the third position (A3s, U3s, C3s and G3s), GC content at third codon positions (GC3s), codon adaptation index (CAI), Codon Bias Index (CBI), effective number of codon (ENC) were determined with CodonW^20^. Relative synonymous codon usage (RSCU) was analyzed with bioPython. KaKs calculator program^21^ with the NG model to calculate the rates of nonsynonymous (Ka), synonymous (Ks), and their ratio (Ka/Ks). When Ks = 0, the value cannot be computed and was represented by *. When Ka = 0 and Ks = 0, the value was represented by NaN. *G. sinensis* was used as a reference.

### Comparative analysis

The genome sequences were initially aligned using MAFFT (v7.310)^22^. The complete chloroplast genomes were compared by the mVISTA program (http://genome.lbl.gov/vista/mvista/submit.shtml). DnaSP (DNA Sequences Polymorphism)^23^ was used to calculate the nucleotide diversity (Pi) of coding, non-coding regions and whole complete chloroplast genomes (all chloroplast genome sequences were adjusted to start with LSC, the step size was set to 200 bp with a window length of 600 bp).

### Phylogenetic analysis

All chloroplast genome sequences were aligned by MAFFT software. The shared CDS genes were extracted by python and aligned using MAFFT. Phylogenetic analysis and tree models was conducted by using the IQTREE software (v2.0.3)^24^. The phylogenetic tree was visualized using ggtree R package (v3.2.1)^25^.

## Results

### Assembly and annotation of the *Gleditsia* chloroplast genome

The seven chloroplast genome of *Gleditsia* genus and two species from *Gymnocladus* genus both showed the typical quadripartite structure of angiosperms, consisting of a large single copy (LSC) region (91,203–91,436 bp) and a small single copy (SSC) region (18,845–19,561 bp), which separated by two inverted repeat (IRA and IRB) regions (26,122–26,619 bp); The chloroplast genome sizes of *Gleditsia* species ranged from 162,746 bp (*G. sinensis*) to 170,796 (*G. delavayi*) (Table S1). The GC content of genomes ranged from 33.9 to 35.64%. *G. sinensis* encoded 84 protein-coding genes, 8 rRNA, 37 tRNA, of which three genes (*ycf1*, *ycf3*, *clpP*) have two introns. *G. fera*, *G. delavayi*, Zhū Yá Zào, and *G. microphylla* encoded 85 protein-coding genes, 8 tRNA genes, and 37 tRNA genes, respectively. *G. microphylla* mutant encoded 84 protein-coding genes, 8 tRNA genes and 37 tRNA genes.

### Analysis of the chloroplast genome structure of *Gleditsia*

**Figure 1:**
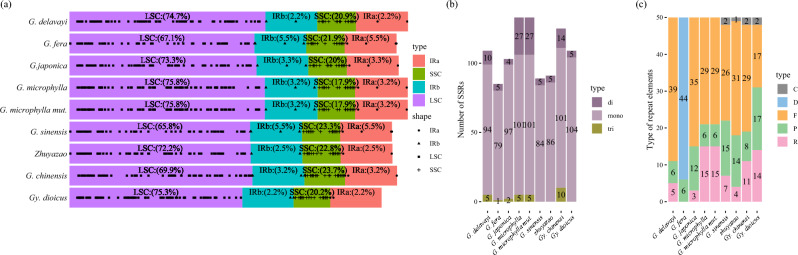
Analysis of SSR sites and repetitive sequences in 9 chloroplast genomes. (**a**): Distribution of SSRs in the *Gleditsia* and two plastomes from *Gymnocladus*; (**b**)*:* Number of different SSRs *loci* types; (**c**): Number of different repeats types. *Note* In a, different shapes represented the position of SSR, and the proportion of text displayed; In (**c**), F: forward repeats, P: palindromic repeats, R: reverse repeats, C: complementary repeats.

Using GCview to visualize the sequence alignment between multiple chloroplast genomes, it was found that the sequence between different species of *Gleditsia* were similar. Through SSR identification, in *Gleditsia* plastomes, the total number of SSRs ranges from 85 to 133 SSRs, while in the *Gymnocladus* it varied from 109 to 125. Moreover, most (65.8–75.8%) of the SSRs in *Gleditsia* and *Gymnocladus* species were located in the LSC. In *Gleditsia*, the IR regions include between 2.2 and 5.5% SSRs loci, while the SSC region included between 17.9 and 23.3% (Fig. 1a). In the *Gymnocladus* sequenced here, 69.9–75.3% of the SSRs were situated in the LSC. A total of 89 SSR sites were detected in *G. sinensis*, including 85 mononucleotide and 4 dinucleotide repeat units. The most abundant repeats were mononucleotide repeats in the *Gleditsia* genus (Fig. 1b). There were 50 repeats in *G. sinensis* (Fig. 1c), which included complementary, forward, palindromic, reverse repeats.

**Figure 2:**
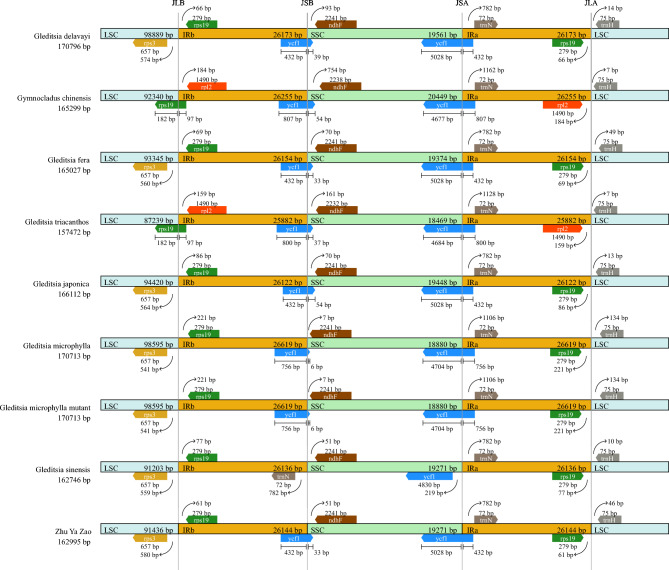
Comparison of the border regions of LSC, IR and SSC. *Note* JLA: junction between LSC and IRa; JLB: junction between LSC and IRb; JSA: junction between SSC and IRa; JSB: junction between SSC and IRb. The numbers above the gene features indicated the distance from the end of the gene to the boundary sites; these features were not scaled.

The chloroplast genome sequence in *Gleditsia* was analyzed using the chloroplast genome of *G. sinensis* as a reference with mVISTA. It was found that the chloroplast genome sequences within the genus *Gleditsia* were highly similar and conserved, with the coding region being more conserved than the non-coding region, and the IR region being more conserved than the SC region. The IR/SC junctions of the chloroplast genome within *Gleditsia* showed similar features (Fig. 2). The lengths of the IR regions in the *Gleditsia* chloroplast genome ranged from 26,122 to 26,619 bp. The *rps3* gene was present in the LSC region in *G. sinensis*, Zhū Yá Zào, *G. fera*, *G. japonica*, *G. delavayi*, *G. microphylla*, and *G. microphylla mut.*, and all IRs contained a gene *rps19*, ranging from 61 to 221 bp from the JLB (junction between LSC and IRb) junction. In the sequenced *Gleditsia* species, the *ndhF* gene was completely present in the SSC and away from the junction, and the *trnH* gene was entirely located in the LSC region. These data suggest that the expansion and contraction of the IR/SC region exhibit similar patterns within *Gleditsia*.

### Codon bias analysis and selective pressures in the evolution

The GC and GC3s content in the codons of the 9 chloroplast genomes studied were both less than 0.5, indicating a preference for A/T bases and A/T-ending codons in *Gleditsia* and *Gymnocladus* chloroplast genomes. We used the CDS of the chloroplast genome to estimate the codon usage frequency of the seven taxa of *Gleditsia*. All 20 amino acids were encoded by codons in the *Gleditsia* chloroplast genome and the synonymous codon usage (RSCU value) values were similar. Of the 29 codons with an RSCU value > 1, only one ended with G (TTG). The codons with an RSCU value < 1, except for ATA and CTA ending in A, ended in C or G. Codon pairs ending with C and G in the *Gleditsia* chloroplast genome had low bias and were non-preferred codons.

**Figure 3:**
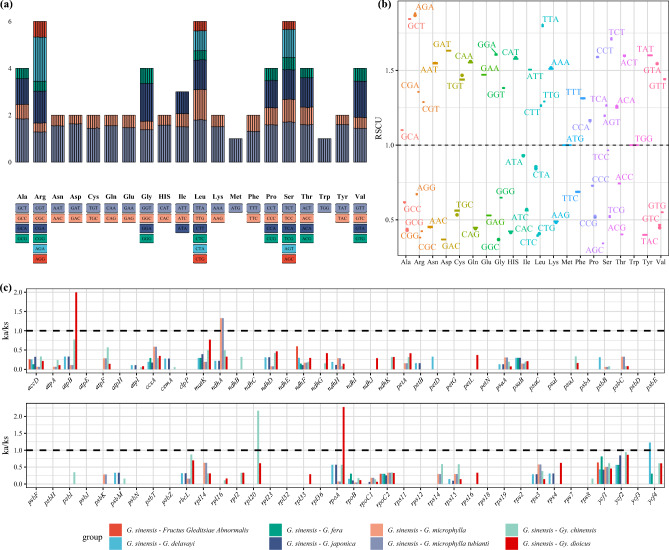
Codon bias analysis and selective pressures in the evolution. (**a**): Codon content of 20 amino acids and stop codons in all protein-coding genes of *Gleditsia* chloroplast genome; (**b**): Distribution of codon preference in *Gleditsia*; (**c**): Ka/Ks values of protein-coding genes of the seven comparative combinations. *Note* In the a, the top panel shows the RSCU for the corresponding amino acids, the colored block which are shown in the below represent different codons; In (**c**), Ka: nonsynonymous; Ks: synonymous.

We analyzed the ka/ks ratio of the 76 unique protein-coding genes in the 9 chloroplast genome (Fig. 3), using *G. sinensis* as the reference, five genes (*rpoA*, *rpl20*, *atpB*, *ndhA*, *ycf4*) were identified under positive selection (Ka/Ks > 1). Ka/Ks ratio of most gene was less than 1.

### Nucleic acid polymorphism analysis

**Figure 4:**
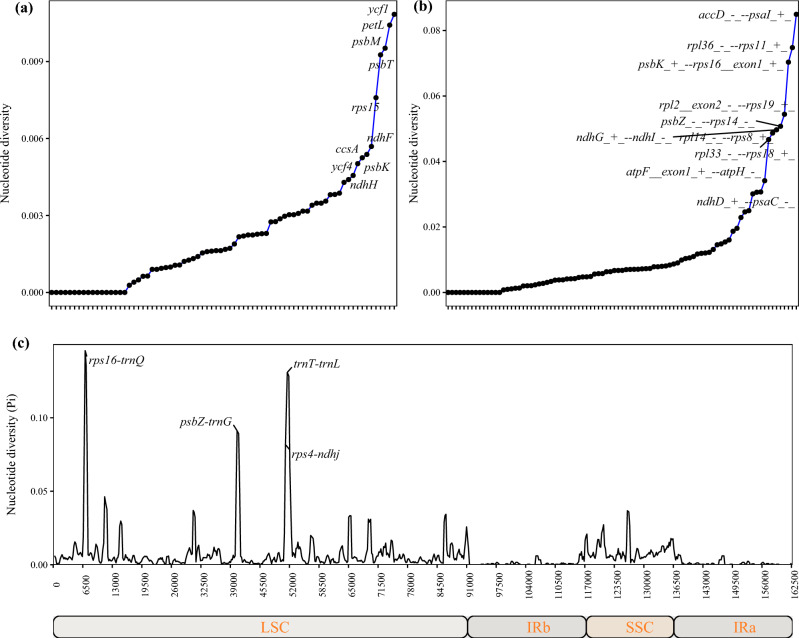
Nucleotide diversity of chloroplast genomes in *Gleditsia*. (**a**): Pi in CDS; (**b**): Pi in intergenic regions; (**c**): chloroplast genome Pi values. *Note* *G. sinensis* was used as a reference genome for comparison, window length: 300 bp, step length: 200 bp; X axis: position of the midpoint of each window; Y axis: Pi of each window.

We conducted an analysis of Pi values to measure the divergence level in protein-coding genes (Fig. 4a), intergenic regions (Fig. 4b) and whole chloroplast genome sequences (Fig. 4c) of the 7 *Gleditsia* species. Taking the common protein coding sequence of *Gleditsia* as the research object*, ycf1* and *petL* were found as mutational hotspots. Through gene sequence alignment and polymorphism analysis using the chloroplast genome of *Gleditsia* as a reference, we found that mutational hotspots occurred in the intergenic regions such as "*rps16*-*trnQ*", "*trnT*-*trnL*", "*ndhG*-*ndhI*", "*rpl32*-*trnL*", etc. The IR region was conserved relative to the SC region.

### Phylogenetic analysis

**Figure 5:**
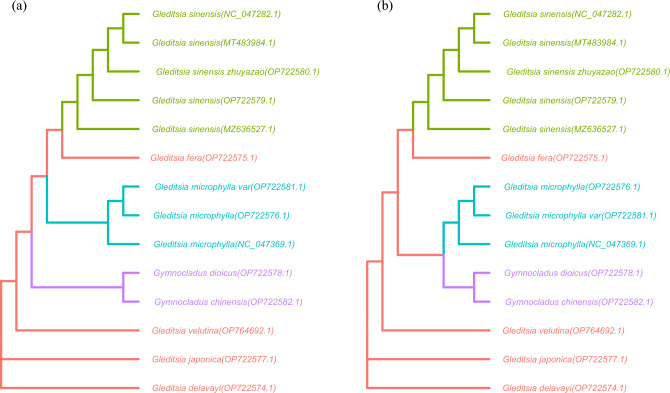
*Gleditsia* phylogenetic tree analysis using the maximum likelihood (ML). (**a**): Phylogenetic analysis based on chloroplast genome sequence; (**b**): Phylogenetic analysis based on shared CDS sequence.

Fourteen chloroplast genome sequences were used for constructing the systematic evolutionary tree, nine of which were provided by this study and five were provided by other studies, the accession numbers can be found at the end of each branch. Based on the full-length chloroplast genome and shared CDS sequences, the optimal model TVM + F + I was calculated by IQTREE according to BIC. Phylogenetic analysis was conducted using the maximum likelihood (ML) based on the full-length chloroplast genome and shared CDS sequences, and the resulting trees (Fig. 5) showed the two datasets produced similar phylogenetic trees with high support and only differed for some nodes' supporting values, the topologies of the ML based on the full-length chloroplast genome and ML based on the shared CDS sequence were essentially consistent. *G. sinensis* and Zhū Yá Zào clustered into a subclade. *G.fera* was most closely related to *G. sinensis.* Two *G. microphylla* (OP722576.1, NC_047369.1) and *G. microphylla* mutant formed a single branch.

## Discussion

The chloroplast genome generally ranges in size from 120 to 160 kb and exhibits a highly conserved structure^26^. The sequencing, assembly, and analysis of chloroplast genomes can identify common features or differences between species, which can be used as DNA barcodes. Seven *Gleditsia* species and two *Gymnocladus* species both have a circular tetrad structure, consisting of one LSC and SSC region, separated by two IR inverted repeat regions, the size of the *Gleditsia* chloroplast genome ranged from 162,746 to 170,907 bp. Most of the SSRs were located in the intergenic areas^27^. Based on SSR identification and examination of their location on the chloroplast genome, it was found that Mononucleotide repeats were the most abundant SSR type in the *Gleditsia* genus. The majority of SSRs (65.8–75.8%) in *Gleditsia* and *Gymnocladus* species were located in the LSC region, which is consistent with previous reports on chloroplast SSRs in other plants^28,29^.

Codon usage of highly expressed genes was selected in evolution to maintain the efficiency of global protein translation^30^. The RSCU values of the CDSs of *Gleditsia* in the present study were similar, the RSCU values of tryptophan and methionine amino acids were 1, they were the only amino acids with no codon bias. There were 29 codons with an RSCU value > 1, only one of which ended with G (TTG); The codons with an RSCU value < 1, except for ATA and CTA ending in A, ended in C or G, the codon pairs ending with C and G in the *Gleditsia* chloroplast genome had low bias, and they were nonpreferred codons. The codons with an RSCU value > 1 were prefer A/T-ending codons in *Gleditsia* genus (Fig. 3b). Six *Euphorbiaceae* plant species^31^ and seven *Miscanthus* species^32^ were biased towards A/T bases and A/T-ending codons. *Quercus* chloroplast genomes prefer A/T-ending codons and avoid C/G-ending codons^33^. Additionally, the Ka/Ks revealed selection pressure on protein-coding genes^34^, Ka/Ks ratios > 1, close to 1, or < 1 indicate that the gene has undergone positive selection, neutral selection, or purifying selection, respectively^35^. The Ka/Ks ratios for the majority (74 of 79) genes were below 1 for the four *Carya* species, indicating that purifying selection were acting on these genes in *C. illinoinensis*^36^. Most of the CDS genes in *Chrysosplenium* had a Ka/Ks ratio range from 0.1 to 0.3, implying strong purification^37^. The average Ka/Ks ratio was 0.17, indicating that the genes in the *Eruca sativa* were subject to strong purifying selection pressures^38^. Purifying selection constantly sweeps away deleterious mutations in population, the purifying selection on most chloroplast genes within *Chrysosplenium* would be evolutionary result of the preservation of the adaptive characteristics of *Chrysosplenium* species^37^. *G. microphylla* is used currently for food, health care products, and cosmetics, as well as for the treatment of various cancers and heart, vascular, and infectious diseases^39^. *G. japonica* pod flat, irregularly twisted; *G. delavayi* distributed only in Yunnan and Guizhou Province, China; *G. fera* distributed gentle slopes, mountain valleys, forests, beside villages, near roads, sunny places, occasionally cultivated, among the species studied, *G. fera* can be divided into fast-growing genotype^5^; *G. australis* seed implantation site is obviously swollen, few fruitless necks^40^. *G. velutina* is endemic to Hunan Province, China, and is a rare and endangered plant under national key protection^41^. Stress-related genes had been positively selected during the evolution through comparative transcriptome analysis of *Gleditsia* genus^42^. In this study, positive selection was acting on five genes (*rpoA*, *rpl20*, *atpB*, *ndhA*, *ycf4*), which were identified under positive selection (Ka/Ks > 1), Ka/Ks ratio of most gene were less than 1, pairwise Ka/Ks analysis showed that most of the chloroplast genes of *Gleditsia* species underwent purifying selection, the purifying selection on most chloroplast genes within *Gleditsia* would be evolutionary result of the preservation of the adaptive characteristics of *Gleditsia* species.

IR region can indicate the distance between species to a certain extent^43^. The highly variable regions can provide useful plastid markers for studies regarding the identification, phylogeny, and population genetics^44^. Using mVISTA to analyze chloroplast genome sequences within the genus *Gleditsia*, coding regions were more conserved than non-coding regions, and IR regions were more conserved than SC regions. Analysis of IR amplification data indicates that expansion and contraction of IR/SC regions show similar patterns within the genus, which is also proved from the polymorphism analysis, in which the IR regions were conserved relative to the SSC and LSC regions, similar to studies in other plants^45^. Mutation hotspots can be used as suitable loci for population genetics and phylogenetic studies. Hypervariable regions can be as candidates for DNA barcode development^46^. DNA barcodes derived from chloroplast genomes will be useful for identifying varieties and resources^12^. DNA barcodes with the largest nucleotide diversity are considered to be the focus of phylogenetic analysis and plant identification^47^. Chloroplast gene sequences (*ndhF* and *rpl16*) are selected to test biogeographic hypotheses, there is a fundamental division of the genus *Gleditsia* into three clades^9^. According to sliding window analysis, *rps16-trnQ*, *rpl32-trnL*, *ndhD-psaC* and *ycf1* showed the greatest variations in *Ilex*^48^. The several non-coding sites (*psbI*–*atpA*, *atpH*–*atpI*, *rpoB*–*petN*, *psbM*–*psbD*, *ndhf*–*rpl32*, and *ndhG*–*ndhI*) and three genes (*ycf1*, *ycf2*, and *accD*) showed significant variation^49^. Positive selection is observed in 14 protein coding genes (*accD*, *ccsA*, *ndhA*, *ndhB*, *psbJ*, *rbcL*, *rpl20*, *rpoC1*, *rpoC*2, *rps12*, *rps18*, *ycf1*, *ycf2* and *ycf4*) in nine species of subfamily Zingiberoideae^50^. Ka/Ks values of three genes *petL*, *rpl20*, and *ycf4* were higher than one in the pairwise comparation of *Galegeae officinalis* and other three *Galegeae* species^51^. Mutational hotspots of shared genes and intergenic spacers of the chloroplast genomes of the *Gleditsia* species were identified. Taking the common protein coding sequence of *Gleditsia* as the research object*, ycf1* and *petL* were found as mutational hotspots. *ycf1* encodes unknown function proteins. The *petL* gene encodes the 3.5 kDa subunit of cytochrome b6/f complex^52^. In other studies, two regions of the plastid gene *ycf1*, *ycf1a* and *ycf1b*, were the most variable loci and of 420 tree species, 357 species could be distinguished using *ycf1b*^53^. The polymorphism of chloroplast genome is useful for evolutionary analysis of *Gleditsia*. Mutational hotspots in *Gleditsia* were found in the intergenic regions such as "*rps16*-trnQ", "*trnT-trnL*", "*ndhG-ndhI*", and "*rpl32-trnL*". *trnK-rps16* (exon2-intron), *trnT-trnL* and *ycf1* are also reported in *Allium*^54^. These hypervariable regions as potential DNA barcode regions for *Gleditsia*.

A genetic distance analysis based on the ISSR genetic diversity revealed that *G. japonica* and *G. delavayi* had a closer genetic relationship^55^. By using the complete chloroplast genomes and shared CDS genes, phylogenetic analysis was performed. The results showed that the two datasets produced similar phylogenetic trees, the relationships of genus were consistent with high support and only differed for some nodes' supporting values. Based on morphology and phylogenetic analysis, *G. japonica* and *G. delavayi* appear most closely related. Zhū Yá Zào is derived from the plant *G. sinensis*, produced by old or injured plants, there was no significant difference in the contents of saponin compounds between *Fructus Gleditsiae abnormalis* and *Fructus Gleditsiae sinensis* by LC-ELSD^7,56^. Li et al.^8^ suggested that Zhū Yá Zào should be a variant of *G. sinensis*. The evolutionary relationship between *G. sinensis* and Zhū Yá Zào was the closest, *G. sinensis* and Zhū Yá Zào clustered into a subclade (Fig. 5). Zhū Yá Zào can be considered a bud mutation of the *G. sinensis*.

Albino phenotypes often occur in nature. In the process of raising seedlings in the greenhouse, a albino mutant plant of *G. microphylla* was obtained (labeled *G. microphylla* mutant), which was characterized by albino whole plant, obvious dwarfing, and natural death after 1–1.5 months of growth. *OsSLC1* is responsible for the seedling-lethal chlorosis phenotype in the rice seedling-lethal chlorosis 1 mutant, loss-of-function of *OsSLC1* affected the intron splicing of multiple group II introns, and especially precluded the intron splicing of *rps16*^57^. The albinism of *Camellia sinensis* cv. *Baiye1* was due to chloroplast dysplasiaand the blocking synthesis of Pchlide a from Mg-proto IX^58^. Deficiency in grana stacking in chloroplasts and inhibition of gene expression related to chloroplast localization may also lead to the production of albino seedlings^59^. By assembling and comparing the chloroplast genomes of the *G. microphylla* mutant and *G. microphylla*, we found that their sequences were completely identical. This suggests that the albino phenotype is not caused by variations in the chloroplast genome, and that the occurrence of the albino phenotype may be due to mutations in chloroplast-related genes involved in splicing or localization functions. This requires further experimental validation in the future.

## Conclusion

In this study, we sequenced and compared the complete chloroplast genomes of seven genotypes from *Gleditsia*. Assembly and annotation of the chloroplast genomes found that *Gleditsia* species chloroplast genomes have a typical circular tetrad structure, the size of the chloroplast genomes ranged from 162,746 to 170,907 bp. Through comparative genomic analysis, most (65.8–75.8%) of the SSRs in *Gleditsia* and *Gymnocladus* species are located in the LSC. The codon pairs ending with C and G in the *Gleditsia* chloroplast genome have low bias which are nonpreferred codons, the genus *Gleditsia* prefer T/A-ending codons and avoid C/G-ending codons. The selection pressure estimation (Ka/Ks ratios) of genes in the *Gleditsia* species showed that *rpoA*, *rpl20*, *atpB*, *ndhA* and *ycf4* were subjected to positive selection, most of the chloroplast genes of *Gleditsia* species underwent purifying selection. The genus *Gleditsia* face relatively weak selection pressure. Mutational hotspots mostly occurred in *"rps16*-*trnQ*", "*trnT*-*trnL*", "*ndhG*-*ndhI*", "*rpl32*-*trnL*" and other intergenic regions in *Gleditsia*. Phylogenetic analysis shows that *G. fera* was most closely related to *G. sinensis, G. japonica* and *G. delavayi* were relatively close, Zhū Yá Zào can be considered a bud mutation of the *G. sinensis.*

### Supplementary Information

Supplementary Information 1.

Supplementary Information 2.

## Data Availability

The datasets generated and analyzed in this study are available in the GenBank of NCBI, and the complete chloroplast genome sequence were available under the accession Number OP722579-OP722582.
