# Supplementary material for: Characterization of the chloroplast genome of Gleditsia species and comparative analysis
Source: Sci Rep. 2024 Feb 21;14:4262. doi: 10.1038/s41598-024-54608-6 (PMC10881578; doi:10.1038/s41598-024-54608-6)
Supplement: Supplementary file 1 — Supplementary Information 1. [file 41598_2024_54608_MOESM1_ESM.docx]

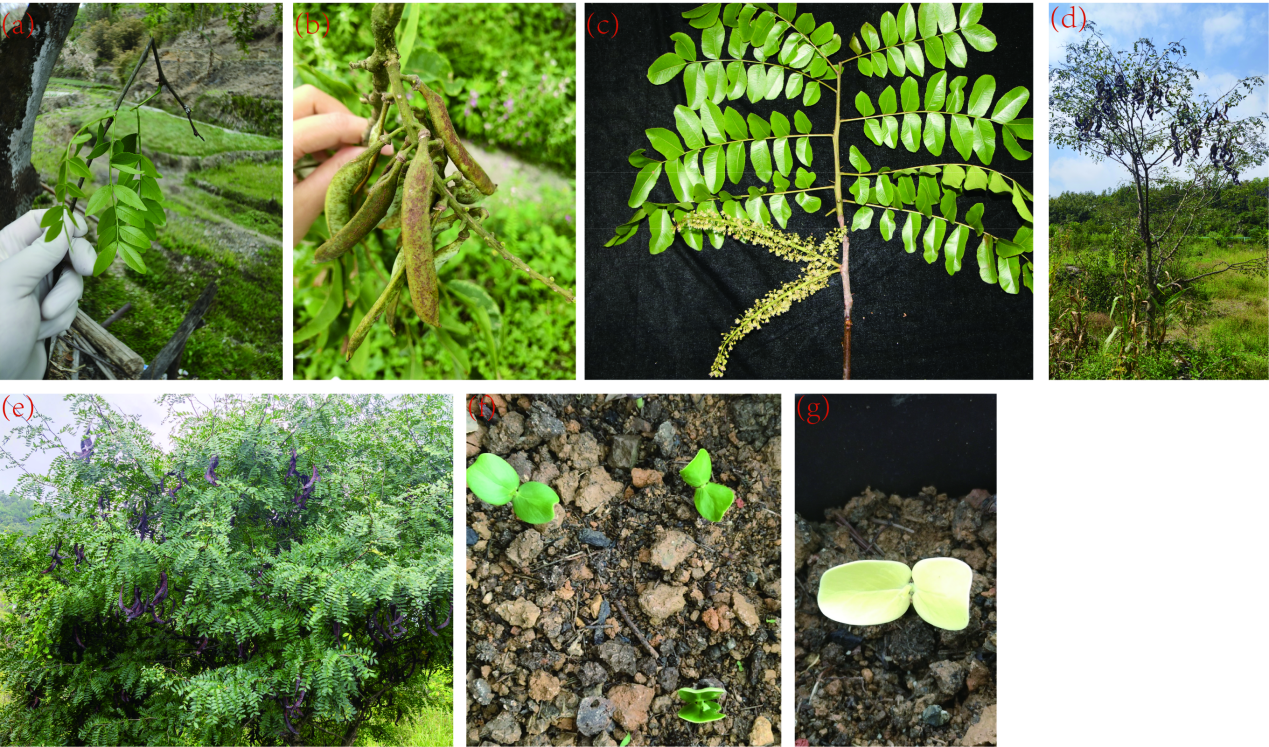


Fig S1. the source of the parent plant or progeny seedlings.

(a):*Gleditsia sinensis*; (b):Zhū Yá Zào (*Fructus Gleditsiae Abnormalis*); (c):*Gleditsia fera*; (d):*Gleditsia japonica* var. *delavayi*; (e):*Gleditsia japonica*; (f):*Gleditsia microphylla*; (g):*Gleditsia microphylla* mutant
