# Supplementary material for: Characterization of the chloroplast genome of Gleditsia species and comparative analysis
Source: Sci Rep. 2024 Feb 21;14:4262. doi: 10.1038/s41598-024-54608-6 (PMC10881578; doi:10.1038/s41598-024-54608-6)
Supplement: Supplementary file 2 — Supplementary Information 2. [file 41598_2024_54608_MOESM2_ESM.docx]

# Supplementary Figures and Tables

**Supplementary Table S1.** Characteristics of assembled chloroplast genome basic structure

| **Species** | **Total size** | **GC%** | **LSC size** | **IR size** | **SSC size** | **Protein coding genes** | **rRNA** | **tRNA** | **NCBI accession** |
| --- | --- | --- | --- | --- | --- | --- | --- | --- | --- |
| *Gleditsia sinensis Lam* | 162746 | 35.64% | 91203 | 26136 | 19271 | 84 | 8 | 37 | OP722579 |
| *Zhū Yá Zào* | 162995 | 35.58% | 91436 | 26144 | 19271 | 85 | 8 | 37 | OP722580 |
| *Gleditsia microphylla* | 170713 | 33.97% | 98595 | 26619 | 18880 | 85 | 8 | 37 | OP722576 |
| *Gleditsia microphylla mutant* | 170713 | 33.97% | 98595 | 26619 | 18880 | 85 | 8 | 37 | OP722581 |
| *Gleditsia japonica* | 166112 | 34.80% | 94420 | 26122 | 19448 | 83 | 8 | 36 | OP722577 |
| *Gleditsia delavayi* | 170796 | 33.90% | 98889 | 26173 | 19561 | 85 | 8 | 37 | OP722574 |
| *Gleditsia fera* | 165027 | 35.17% | 93345 | 26154 | 19374 | 85 | 8 | 37 | OP722575 |
| *Gymnocladus chinensis* | 165299 | 34.96% | 92340 | 26255 | 20449 | 84 | 8 | 37 | OP722582 |
| *Gymnocladus dioicus* | 157472 | 36.36% | 87239 | 25882 | 18469 | 84 | 8 | 36 | OP722578 |
